# Supplementary figures and images for: Different Patterns of Care and Survival Outcomes in Transplant-Centre Managed Patients with Early-Stage HCC: Real-World Data from an Australian Multi-Centre Cohort Study
Source: Cancers (Basel). 2024 May 22;16(11):1966. doi: 10.3390/cancers16111966 (PMC11171392; doi:10.3390/cancers16111966)

## Slide 1
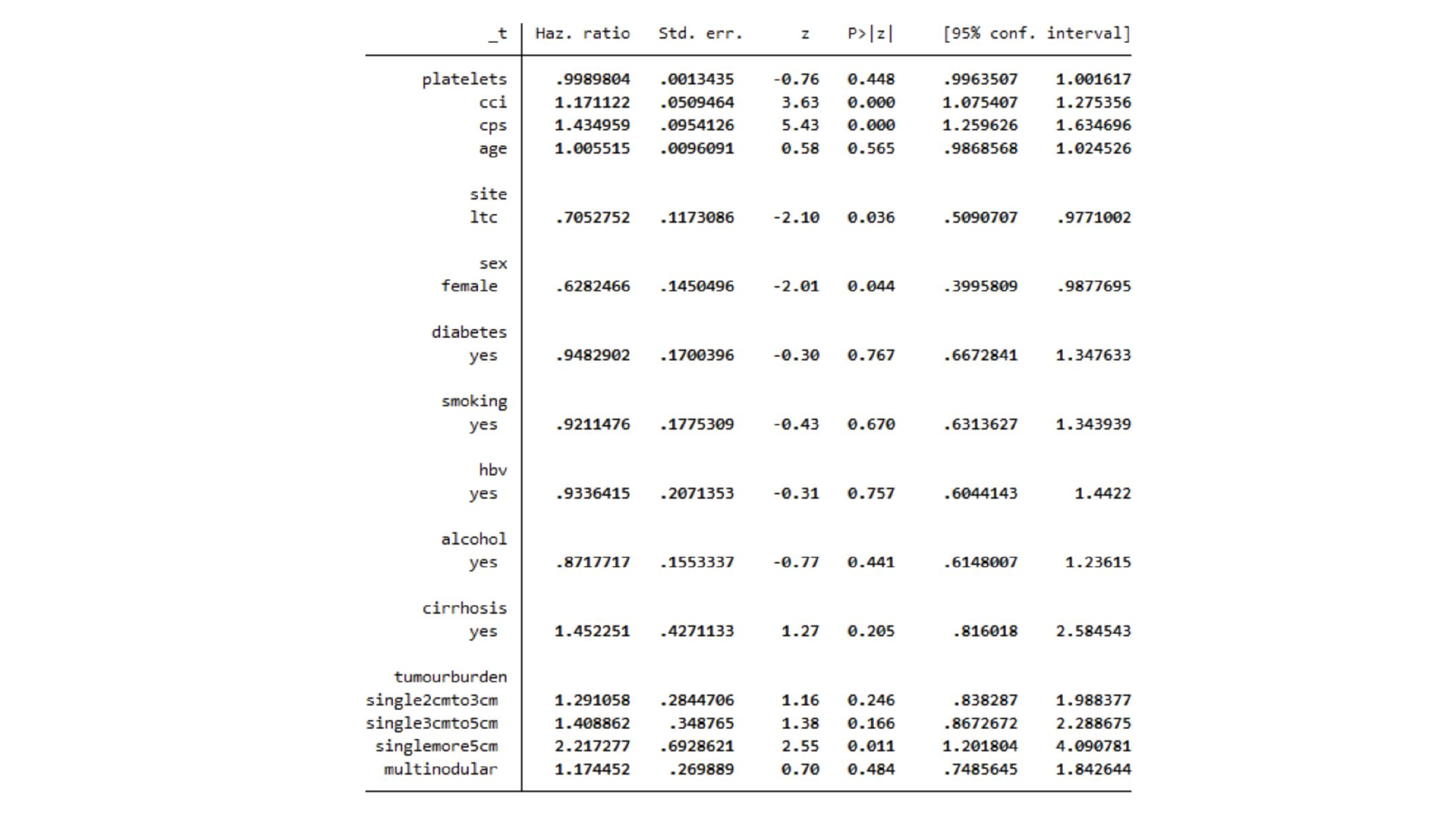

## Slide 2
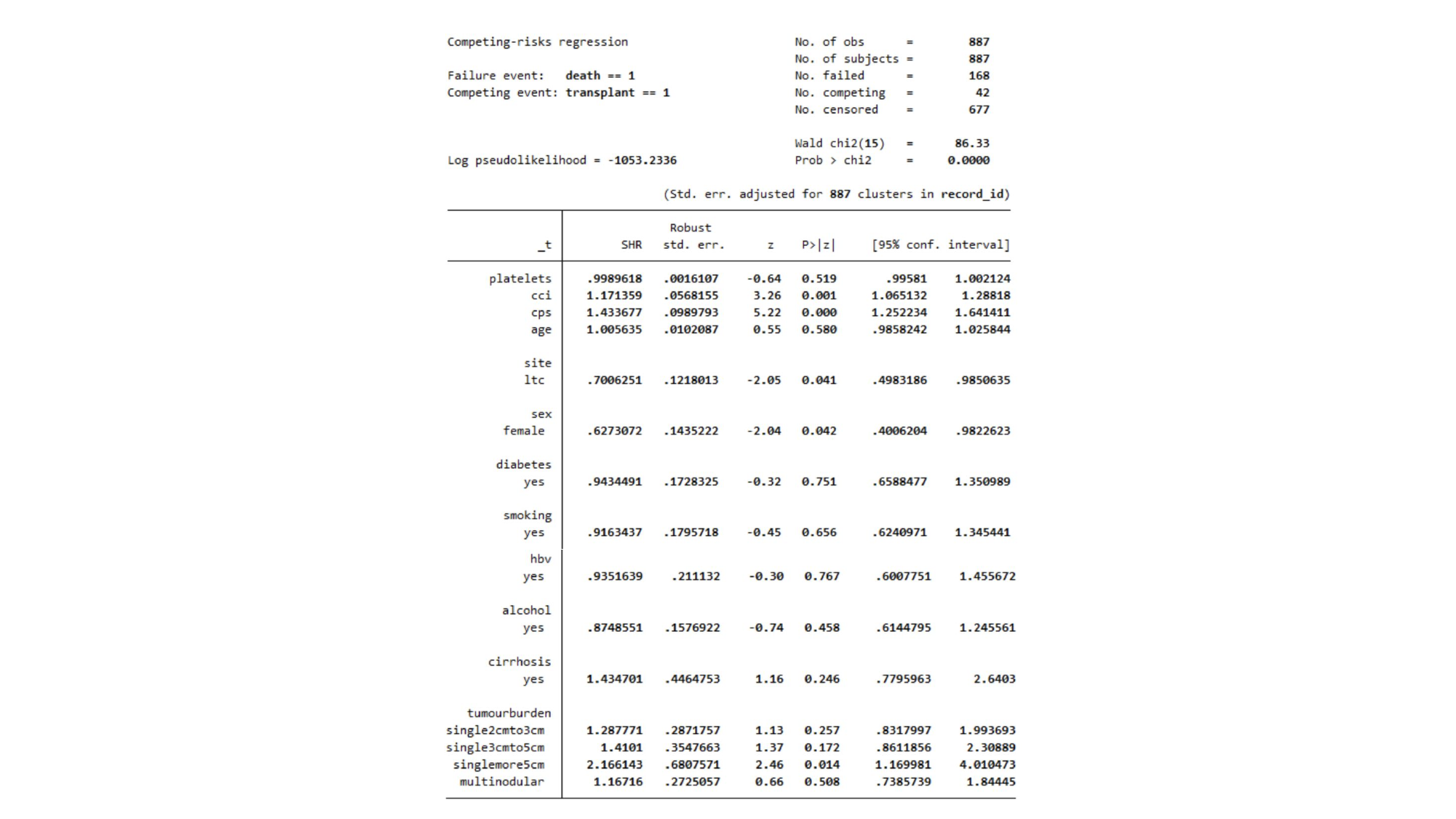

Supplement: Supplementary file 1 [file cancers-16-01966-s001.zip › cancers-3020325-supplementary.pptx]
